# EGFR p.V774M/p. the S768I Compound Mutation in NSCLC Is a Good Prognostic Marker for IPHC‐ and Furmonertinib‐Based Treatment: A Case Report

**DOI:** 10.1002/cnr2.70444

**Published:** 2025-12-30

**Authors:** Yanan Wang, Guanying Ren, Zizheng Song, Ling Hu, Weiqi Li, Xiaolei Wang

**Affiliations:** ^1^ Department of Medical Oncology Affiliated Hospital of Hebei University, Hebei Key Laboratory of Cancer Radiotherapy and Chemotherapy Baoding Hebei China

**Keywords:** compound mutation, EGFR, furmonertinib, IPHC, NSCLC

## Abstract

**Background:**

Intrapleural perfusion hyperthermic chemotherapy (IPHC) can be used to select specific lung cancer cells of a certain genotype. Some genetic mutations, especially epidermal growth factor receptor (EGFR) mutations, are potential markers for EGFR or other targeted therapies. Here, we report a unique case of a patient with EGFR p.V774M/p. S768I mutations that were associated with prolonged stable disease after treatment with IHPC, especially following furmonertinib‐based treatment.

**Case:**

A 47‐year‐old Chinese female patient was admitted to a regional cancer hospital and presented with malignant pleural effusion (MPE). The TNM and clinical stages were identified as T2aN2M1c and IVB, respectively. The TPS value is 40%. IHP combined with chemotherapy was then carried out to remove MPE. For first‐stage treatment, three cycles of afatinib‐based adjuvant chemotherapy beginning in September 2022 were given, and the degree of pleural effusion on the left side of the chest was not reduced. The best response (BOR) assessment of the local lesion was a partial response (PR). Furmonertinib‐based adjuvant monotherapy was performed from May 2023 to July 2024. The BOR evaluation results during the monotherapy courses were all judged as SD.

**Conclusion:**

The EGFR p.V774M/p.The S768I mutation contributed to improving the efficiency of furmonertinib‐based therapy for NSCLC.

## 1 Introduction

Nonsmall cell lung cancer (NSCLC) is the most common type of lung cancer, accounting for 85% of all cancers [1]. Owing to the lack of obvious symptoms and clear biomarkers, one‐third of NSCLC patients are usually diagnosed at advanced stages, and the prognosis is generally poor [2]. In recent years, malignant pleural effusion (MPE) has been regarded as an important factor affecting the efficiency of several types of pharmaceutical therapy [3]. Intrapleural perfusion hyperthermic chemotherapy (IPHC) is a locoregional therapeutic strategy that combines the cytotoxic effects of chemotherapy with the synergistic antitumor activity of hyperthermia. It involves the circulation of heated chemotherapeutic agents within the pleural cavity, aiming to achieve higher local drug concentrations and enhanced tissue penetration while minimizing systemic exposure [4]. For advanced NSCLC patients with MPE, IPHC and subsequent targeted therapy involving driver genes significantly prolong patient progression‐free survival (PFS) [5]. The response to such treatment varies for different individuals [6].

Some mutations in oncogenes have been identified as targets for several small‐molecule drugs, including epidermal growth factor receptor (EGFR), anaplastic lymphoma kinase (ALK), and c‐ROS oncogene 1 (ROS1) [7, 8, 9]. Given its superior efficacy and lower toxicity than chemotherapy, targeted therapy has already been recommended as the first‐line treatment for advanced NSCLC in recent decades [10]. Molecules that target a series of EGFR mutations are normally developed as drugs. In recent decades, several successful EGFR tyrosine kinase inhibitors (EGFR‐TKIs) have been developed and have significantly improved the prognosis of NSCLC patients with EGFR mutations [11]. However, 50% of Asian patients and 80% of Western patients cannot benefit from EGFR‐targeted treatments unless they present with EGFR‐sensitizing mutations [12].

The predominant mutations in EGFR, p.L858R and 19del, are highly frequently detected in NSCLC cohorts and are called classical mutations [13]. According to Chinese data, some mutations in exons 18–21, such as the well‐known mutations exon20ins, exon18 G719X, exon20 S768I, and exon21 L861Q, are called rare mutations [14]. These mutations account for approximately 11.9% of the EGFR mutations in the Chinese cohort [15]. First‐line EGFR‐TKIs lack a clear recommended dosage and clinical efficiency evaluation for patients with rare mutations [16]. In recent years, several alternative treatment methods, such as second‐ and third‐generation irreversible inhibitors, have been investigated for treating advanced patients with rare mutations [17]. However, the clinical benefit cannot be evaluated due to the limited number of clinical subjects.

Some rarely detected EGFR mutations play a significant role in the development and progression of various tumors, particularly NSCLC. In a long‐lasting case of lung adenocarcinoma, the EGFR V774M/L833V compound mutation improved the response to almonertinib treatment [18]. In NSCLC, the EGFR p.S768I mutation, located in exon 20, is a rare but clinically relevant alteration. Molecular dynamics simulations have revealed that the S768I mutation contributes to increasing the structural flexibility of the EGFR protein and enhances its tendency to adopt an active conformation [19]. In a clinical case report, TP53 mutations combined with EGFR S768I/V774M led to a lack of response to targeted drugs such as famitinib and sunvozertinib [20].

In a previous study, several genetic biomarkers were reported to predict a specific population who would benefit from IPHC treatment [17]. However, the associations between rare genetic biomarkers and IPHC treatment are still unclear. Here, we present a patient harboring the EGFR p.V774M//p. S768I comutations who can benefit from good efficacy under IPHC and subsequent targeted therapy. We also show that hyperthermic chemotherapy can select specific EGFR mutation‐positive lung cancer cell lines and benefit from fumatinib‐based targeted therapy.

## 2 Diagnostic Assessment and Analysis Methods

### 2.1 Imaging Studies

A whole‐body positron emission tomography‐computed tomography (PET‐CT) scan was performed for initial staging. Subsequent disease assessments were primarily based on contrast‐enhanced computed tomography (CT) of the chest and abdomen and magnetic resonance imaging (MRI) of the brain.

### 2.2 Pathological Diagnosis

Pathological confirmation of lung adenocarcinoma was obtained through histological examination of both pleural effusion cell blocks and tissue samples from a bronchoscopic biopsy.

### 2.3 Immunohistochemistry (IHC)

IHC staining was conducted on the biopsy samples. In brief, the 4 μm sections were incubated with primary antibodies. The slices were subsequently washed three times in 1× phosphate‐buffered saline (PBS) with 1% Triton. The nuclei were then counterstained with 4′,6‐diamidino‐2‐phenylindole (DAPI). Images were captured with an I‐View Detection Chemistry system (Ventana Medical Systems, Tucson, AZ). The tumor cells were positive for CK7, NapsinA, and TTF‐1 and negative for P40 and CK5/6, supporting a diagnosis of lung adenocarcinoma. Programmed death‐ligand 1 (PD‐L1) expression was evaluated via the tumor proportion score (TPS) via PD‐L1 IHC 22C3 pharmDx (Agilent Technologies, Carpinteria, CA).

### 2.4 Molecular Profiling

Next‐generation sequencing (NGS) was performed on DNA extracted from the pleural effusion sample to identify mutations in a targeted gene panel (the probe sets were designed by Burning Rock Biotech and synthesized by Agilent). The panel was designed according to the information of the OncoKB (Burning Rock Biotech, PMID: 28890946). The proper DNA fragment was harvested with a QIAquick Gel Extraction Kit (Cat No. 28704, Qiagen, USA). The DNA integrity and concentration were determined via a Lab‐on‐a‐Chip‐System Bioanalyzer 2100 (Agilent, USA). PCR was used to amplify the captured DNA region. The sequencing library was prepared with the NEBNext Ultra II DNA Library Prep Kit (Cat NO. NEB#E7645L, NEB, USA). The library was then sequenced via the HiSeq X Ten platform (Illumina, USA). FastQC (v0.10.0, http://www.bioinformatics.babraham.ac.uk/projects/fastqc/) and MuTect (v1.1.4, http://www.broadinstitute.org/cancer/cga/mutect) were then employed to process the raw sequence data. The annotation of the mutations was performed according to the National Comprehensive Cancer Network (NCCN) Guidelines (https://www.nccn.org/) and OncoKB.

## 3 Best of Response (BOR) Assessment Method

The BOR was assessed using the Response Evaluation Criteria in Solid Tumors (RECIST) version 1.1. Tumor response was classified into one of the following categories: complete response (CR), partial response (PR), stable disease (SD), or progressive disease (PD). Evaluations were conducted based on contrast‐enhanced CT scans of the chest and abdomen, MRI of the brain, and PET‐CT when necessary. All imaging assessments were carried out at baseline and following every two cycles of treatment, with BOR being defined as the best response documented from the commencement of treatment until disease progression or the start of subsequent therapy.

## 4 Case Description

### 4.1 Patient Information, Primary Concerns, and Symptom History

**FIGURE 1:**
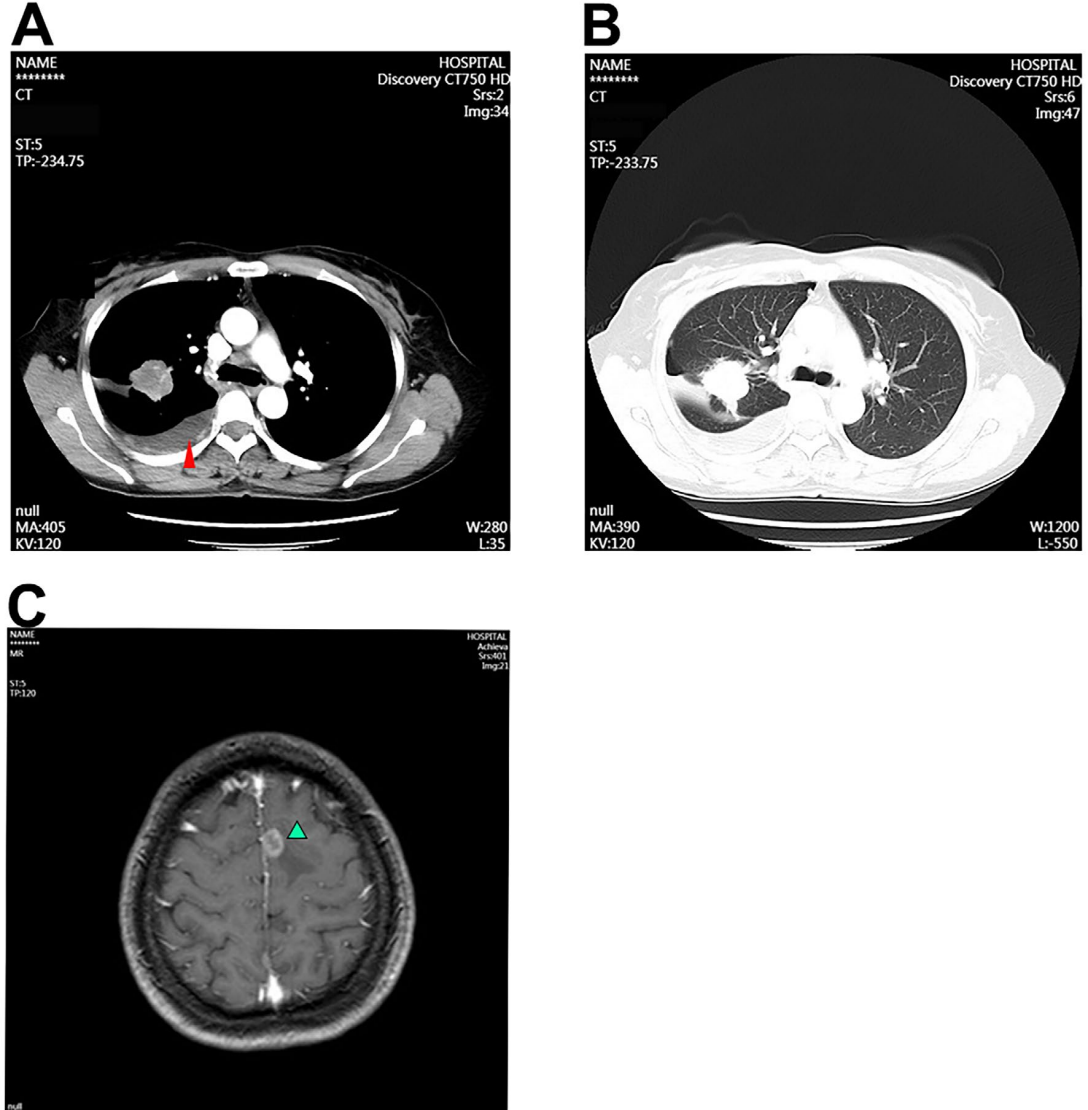
Enhanced CT and head MRI examinations of the patient's chest were performed to assess disease status and progression before treatment. (A, B) The image was captured on July 26, 2022. The high‐density arc‐shaped shadows indicate intrapleural hyperthermic perfusion (red arrowhead). (C) Head MR image of the brain. The green arrows indicate the metastatic focus.

A 49‐year‐old Chinese female who was a nonsmoker presented with a 1‐month history of dyspnea, chest tightness, and mild hemoptysis in July 2022. She reported worsening symptoms in the supine position and occasional productive cough with white sputum. Initial evaluation at a local hospital revealed right‐sided pleural effusion and a suspected right lung mass. A chest CT scan on July 26, 2022, confirmed right pleural effusion, pneumothorax, pulmonary atelectasis, and multiple pulmonary nodules (Figure 1A,B). She was subsequently referred to the Affiliated Hospital of Hebei University for further management.

### 4.2 Clinical Findings and Diagnostic Assessment

**FIGURE 2:**
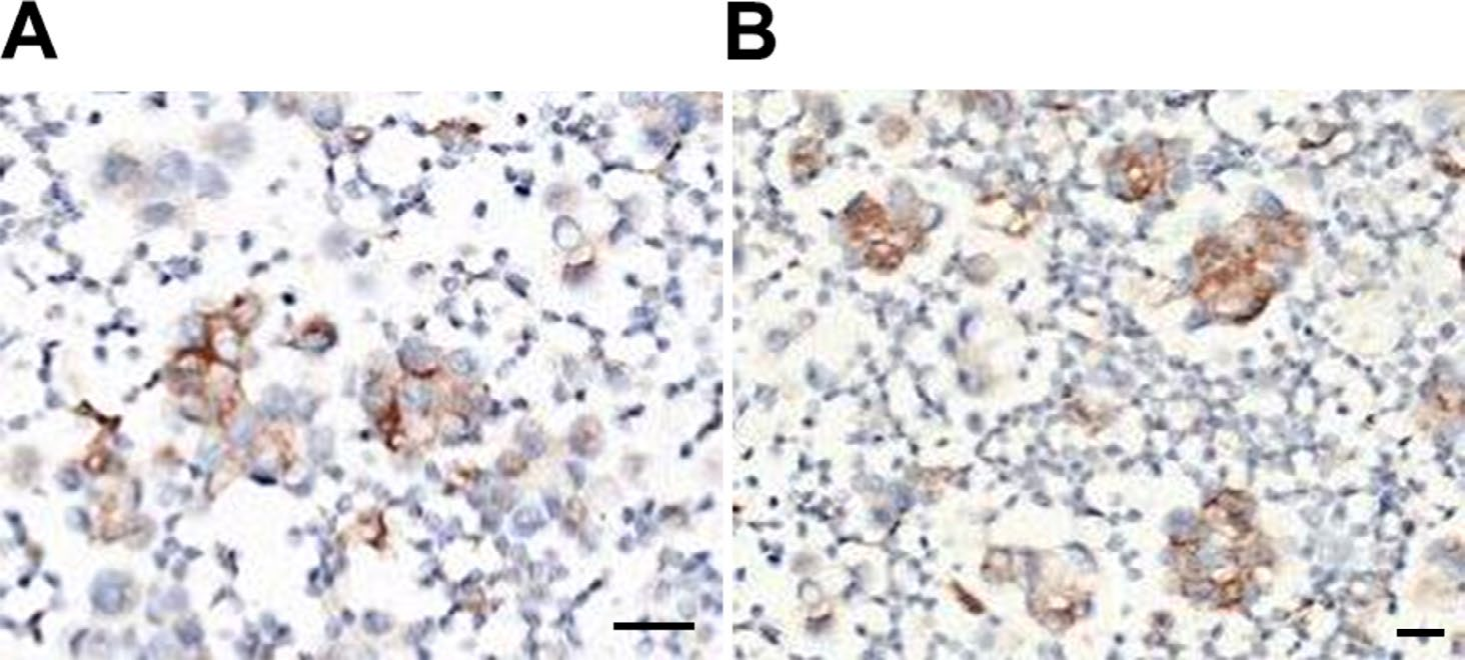
IHC staining of PD‐L1 in lung adenocarcinoma slices from thoracic puncture tissue biopsy. (A) TPS percentage; (B) CPS. Scale bar = 100 μm.

On admission, physical examination was unremarkable except for decreased breath sounds on the right side. Next, whole‐body PET‐CT and thoracic puncture tissue biopsy examinations were performed. She was then diagnosed with peripheral lung adenocarcinoma in the right upper lobe, multiple lung nodules, pleural thickening, and mediastinal and hilar lymphadenopathy. No distant metastasis to the bone, liver, or mediastinal lymph nodes was observed. Head enhancement MRI revealed bilateral occipital and left frontal lobe metastases (Figure 1C). The clinical stage was determined to be T2aN2M1c stage IVB. This indicates the most advanced stage of the tumor. Pathological examination of the pleural effusion fluid and bronchoscopic biopsy confirmed lung adenocarcinoma. The IHC results were positive for CK7, NapsinA, and TTF‐1 and negative for P40 and CK5/6. PD‐L1 testing revealed a TPS of 40% (Figure 2A,B). This TPS score potentially indicates a positive signal for immune therapy, while efficient treatment strategy selection also depends on the mutational status of the cancer‐driven gene [21]. NGS of pleural effusion‐derived DNA identified an EGFR p.S768I mutation with an allele frequency of 9.52%. The other mutational abundances of the targeted genes are as follows: EGFR p.S768I accounts for 9.52%, BMPR1A c.409_418dup accounts for 5%, and RB1 c.1389 + 5G>T accounts for 2.23%. No clear clinical meaning was found for BMPR1A c.409_418dup and RB1 c.1389 + 5G>T. After the recurrence of right pleural effusion in May 2023, a repeat liquid biopsy in May 2023 detected a new EGFR p.V774M mutation (9.37%) in addition to the preexisting p.S768I mutation.

## 5 Therapeutic Interventions and Outcomes

**FIGURE 3:**
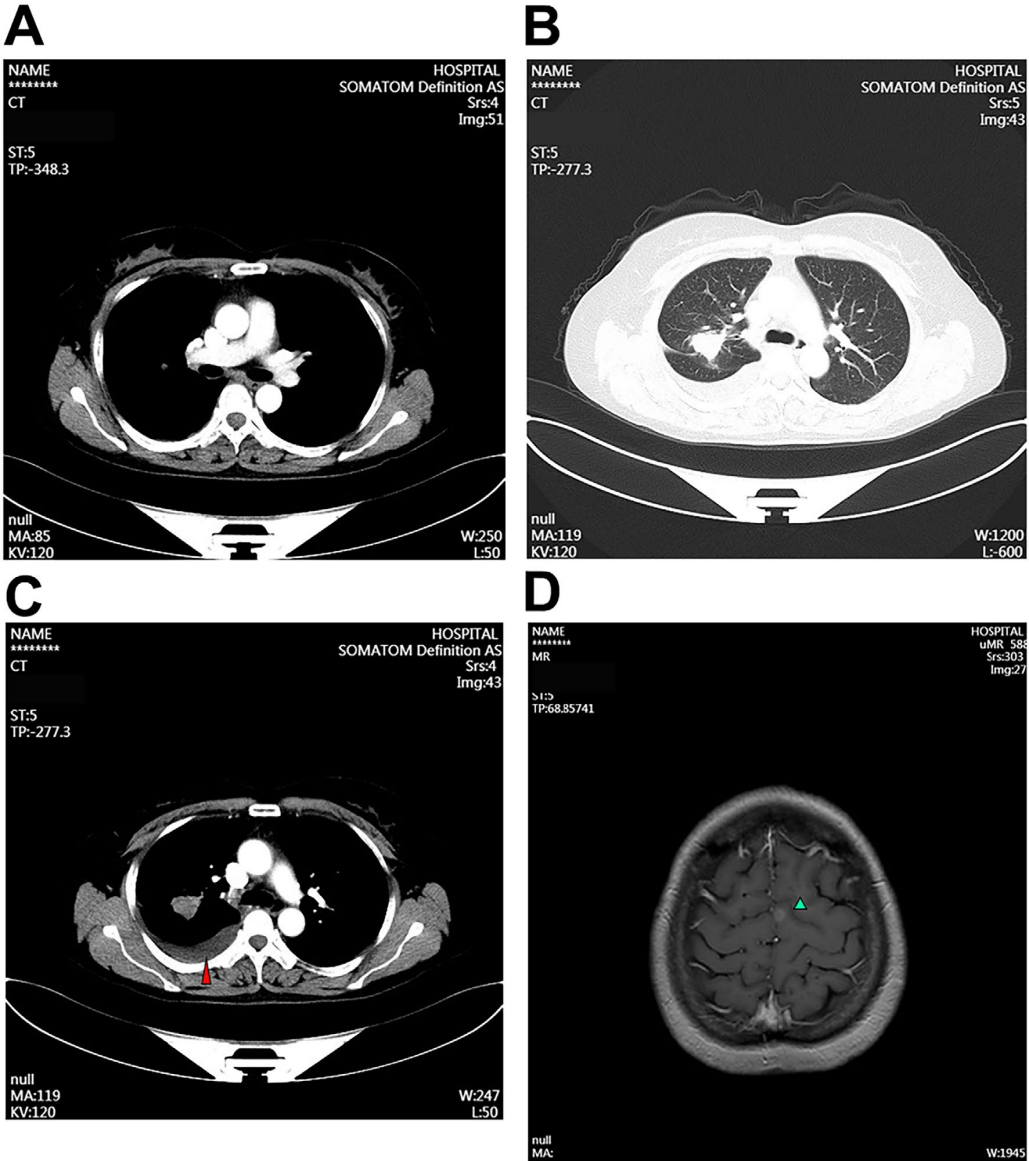
Enhanced CT and head MRI examinations of the patient's chest were performed to assess disease status and progression. (A, B) CT image captured on July 18, 2022. (C) CT image captured on May 5, 2023. The high‐density arc‐shaped shadows indicate intrapleural hyperthermic perfusion (red arrowhead). (D) Head MR image of the brain. The green arrows indicate the metastatic focus.

**FIGURE 4:**
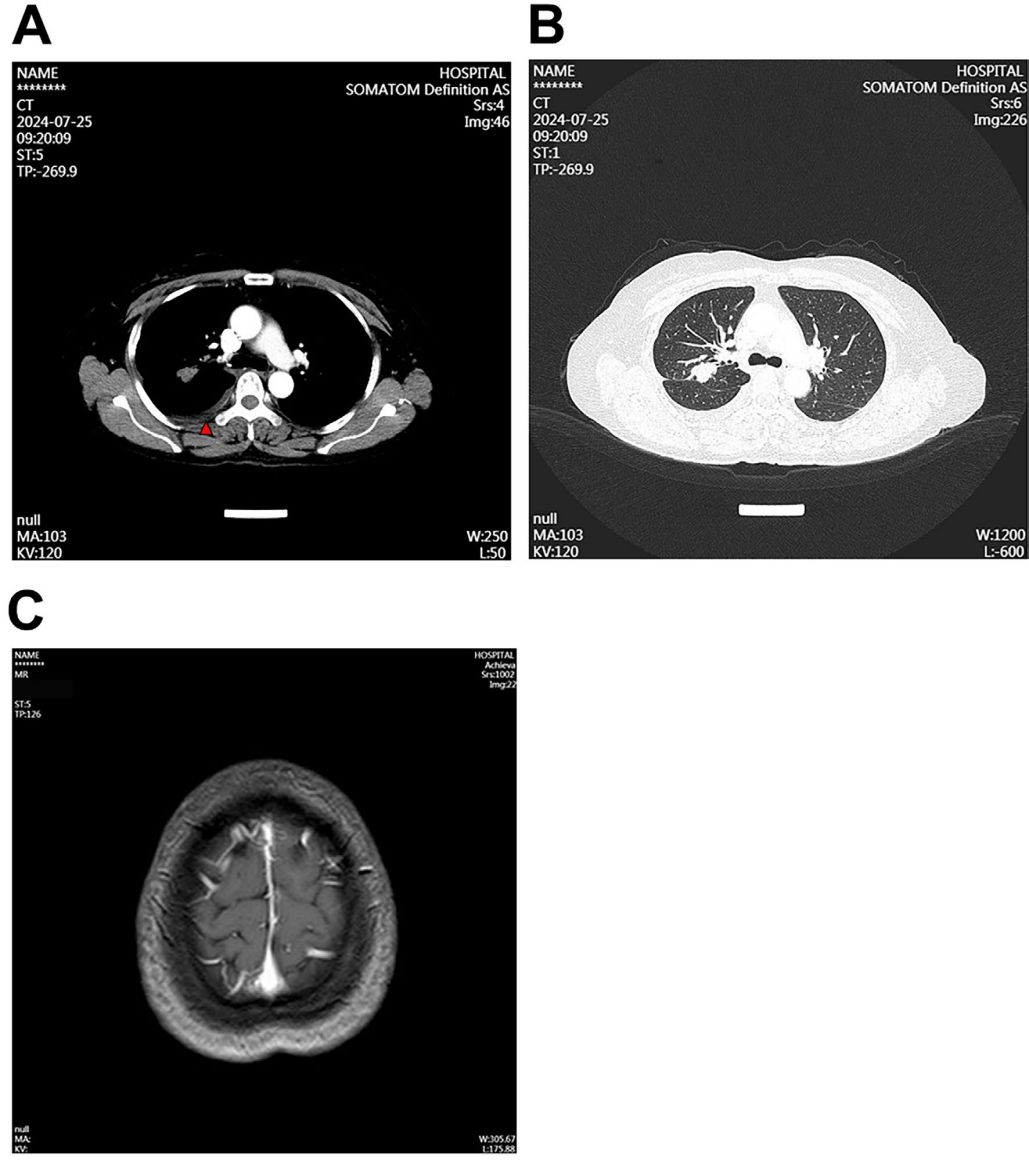
Enhanced CT and head MRI examinations of the patient's chest were performed to assess disease status. (A, B) A CT image was captured on July 25, 2024. The high‐density arc‐shaped shadows indicate intrapleural hyperthermic perfusion (red arrowhead). (C) Head MR image of the brain.

From August 4 to August 18, 2022, the patient underwent four cycles of cisplatin‐based IPHC. Each course lasted for 5 days. After IPHC treatment, the MPE volume was significantly reduced, and little MPE was observed in the CT image (Figure 3A,B). She then received oral afatinib (40 mg/day) combined with intravenous bevacizumab (15 mg/kg every three weeks) beginning in September 2022. Follow‐up imaging in September 2022 revealed a PR with reduced primary lung lesions and brain metastases. However, by May 2023, CT imaging revealed recurrence of right pleural effusion, suggesting possible resistance to afatinib (Figure 3C,D). The PR assessment of the local lesion was evaluated by the BOR criteria. No other abnormal clinical indices were observed after the final course of treatment. Six courses of the third‐generation TKI furmonertinib‐based targeted therapy, which replaces afatinib from May 6, 2023, to July 16, 2024, were then performed. The regimen strategy for each course was consistent with that for furmonertinib 80 mg/day, and bevacizumab was administered at 15 mg/kg once every three weeks. Enhanced chest CT was performed to evaluate the clinical outcome after the treatment course on July 25, 2024. Compared with the CT results of May 5, 2023, the volume of MPE in the lung mass and lymph nodes was reduced, and the number of brain metastases was dramatically reduced (Figure 4A–C).

### 5.1 Follow‐Up and Current Status

After multiple cycles of this regimen, follow‐up imaging in July 2024 demonstrated a marked reduction in pleural effusion, lung lesions, and brain metastases. The BOR was evaluated as SD, and no significant treatment‐related adverse events were reported. The PFS exceeded 10 months as of the cut‐off date. As of the last follow‐up in July 2024, the patient remained clinically stable. She reported good quality of life, with no new symptoms or distant metastases. She continues on furmonertinib and bevacizumab maintenance therapy with regular outpatient monitoring.

## 6 Discussion

The mutation spectrum of cancer‐driven genes has an important effect on the efficiency of TKIs [22]. To date, three generations of EGFR‐TKIs have been developed to treat NSCLC. In this case, resistance to afatinib was observed in a patient with EGFR p.S768I after receiving the first course of IPHC, and the second EGFR p.V774M compound mutation was detected. The second course of IPHC was subsequently performed, and the treatment strategy was changed to the third‐generation agent furmonertinib. Finally, longer PFS and a stable BOR stage were observed. The most significant finding is the marked and durable response to furmonertinib after progression on afatinib, resulting in a PFS of over 10 months. This finding suggests that the p.V774M/p.S768I compound mutation may confer sensitivity to furmonertinib, even in the context of prior TKI resistance. However, the management of such rare mutations remains challenging owing to the absence of standard treatment guidelines and the limited understanding of their structural and functional impact on drug binding.

As first‐line drugs for treating EGFR‐mutated NSCLC, afatinib and the third‐generation TKI osimertinib have been shown to be feasible for treating NSCLC patients with several active mutation sites, especially T790M‐positive EGFR mutation‐positive patients [23]. Except for deletions in exon 19 and L858R in exon 21, which are called “classic” EGFR mutations, the remaining mutations are rare mutations, such as G719X, S768I, and L861Q [24]. The major rare mutations are located in exons 18–25 of the EGFR gene [23]. Several EGFR‐TKIs have been approved for treating rare EGFR mutations [25]. Given the lack of patients and related cell lines with rare mutations, the detailed pharmacological mechanism and specific biological characteristics of these rare EGFR mutations cannot be further investigated. In a previous study, compounds with rare mutations were associated with shorter OS than those with single mutations in the NSCLC cohort [26]. For example, patients with the p.S768I mutation were also reported to be sensitive to afatinib [27]. Furthermore, afatinib has been approved for treating S768I, L861Q, and G719X rare EGFR point mutations by the FDA [28]. However, the other genetic mutation background of the patient and subsequent treatment strategy play important roles in determining the clinical outcome [29].

In the past two decades, several third‐generation agents (osimertinib, aumolertinib, and furmonertinib) have been developed and revolutionized the therapeutic strategy for NSCLC patients with EGFR mutations [30]. The third‐generation TKI osimertinib has been reported to be effective in patients with G719X compound rare mutations in a meta‐analysis [27]. Compared with the other two agents, furmonertinib has better efficacy. Furmonertinib has demonstrated a distinct advantage in treating patients with classical EGFR mutations, particularly L858R and T790M mutations [27]. However, the role of furmonertinib in treating patients with rare EGFR mutations has not often been reported. Zhao et al. reported that furmonertinib can prolong the PFS period of patients with rare mutations and overcome resistance to osimertinib and afatinib [31]. Another study reported that furmonertinib (160 mg/day) can efficiently treat patients harboring uncommon mutations [32]. In this case, furmonertinib efficiently prolonged the PFS of patients with p.V774M/p.S768I EGFR mutations. This potentially provides a new prerequisite for choosing an EGFR‐TKI. Compared with another report in which the EGFR V774M/L833V compound mutation was beneficial for almonertinib treatment, both cases suggested that the EGFR p.V774M mutation may be a positive signal for third‐generation EGFR‐TKI agents [18].

The mutation site p.V774M is located on the C‐terminal inner surface of the a‐C helix of the EGFR molecule. The mutations in this domain belong to the P‐loop and a‐C helix compression type or the PACC type. Structural studies suggest that such mutations can reduce the volume of the ATP‐binding pocket and confer resistance to osimertinib [33]. Another rare mutation site in this patient was p.S768I EGFR, which is an EGFR exon 20 mutation. There are some conflicting clinical reports of tumors harboring p.S768I and its sensitivity to various EGFR‐TKIs [34, 35, 36]. Some reports have suggested that different compound comutations with p.S768I may be the reason for the conflicting results [37]. p.V774M/p. S768I EGFR compound mutations have been reported only to be nearly complete with respect to osimertinib [38]. In a similar case report, TP53 mutations combined with EGFR S768I/V774M led to a lack of response to targeted drugs such as famitinib and sunvozertinib [20]. Conversely, our patient with the S768I/V774M mutation in EGFR can benefit from furmonertinib treatment without causing TP53 mutations.

Furthermore, the role of IPHC in sensitizing tumors to subsequent targeted therapy should not be overlooked. Research has indicated that IPHC may selectively affect tumor cells with certain kinase domain mutations [39]. Specifically, IPHC has been shown to be an effective treatment for patients with EGFR kinase domain mutation‐positive lung cancer [22]. In an in vitro animal model, IPHC enhanced antitumour efficacy in in vitro models of drug‐resistant lung tumors [40]. The associations between IPHC and specific genotypes have also been reported in other cancer studies [41]. In our case, the significant reduction in MPE after IPHC may have created a favorable tumor microenvironment for subsequent furmonertinib efficacy, although this hypothesis requires further investigation. This finding indicates at least two facts: (1) the IPHC may select or cause new mutations; (2) the p.V774M/p. S768I mutation may be a drug resistance factor for afatinib and a sensitivity factor for famitinib. However, the potential mechanism of drug sensitivity and resistance was not fully understood in this study. Further investigations are still needed in the future. Another limitation of our study is its nature as a single case report. Although our findings are promising, the generalizability of the conclusions is limited. Further prospective studies and larger cohort analyses are needed to validate the efficacy of furmonertinib in patients with p.V774M/p. S768I and other rare compound mutations.

## 7 Conclusion

In summary, we present a case of advanced NSCLC with a rare EGFR p.V774M/p. S768I compound mutation that responded favorably to a treatment sequence of IPHC followed by furmonertinib. Our results suggest that this compound mutation may be a potential biomarker for furmonertinib sensitivity. This case adds new evidence supporting the clinical utility of furmonertinib in managing rare EGFR compound mutations, especially after failure of prior TKIs.

## Author Contributions

All the authors made substantial contributions to the study conception and design, acquisition of the data, or analysis and interpretation of the data; Yanan Wang and Guanying Remade equally made substantial contributions to the study conception and design, acquisition of the data, or analysis and interpretation of the data. Zizheng Song and Ling Hu collected the samples and clinical data. Weiqi Li and Xiaolei were involved in the data analysis. Zizheng Song and Ling Hu contributed to the review and revision of the manuscript. All the authors read and approved the final manuscript.

## Funding

This study was supported by Medical Science Research Project of Hebei (Project No. 20240587).

## Ethics Statement

The present study was approved by the Ethics Committee of the Affiliated Hospital of Hebei University (approved number 20220923). The research was carried out in accordance with the World Medical Association Declaration of Helsinki. Written informed consent was obtained from the patients who participated in the study.

## Conflicts of Interest

The authors declare no conflicts of interest.

## Data Availability

The data that support the findings of this study are available on request from the corresponding author. The data are not publicly available due to privacy or ethical restrictions.
